# Beyond Self-Report: A Review of Physiological and Neuroscientific Methods to Investigate Consumer Behavior

**DOI:** 10.3389/fpsyg.2018.01655

**Published:** 2018-09-07

**Authors:** Lynne Bell, Julia Vogt, Cesco Willemse, Tim Routledge, Laurie T. Butler, Michiko Sakaki

**Affiliations:** ^1^School of Psychology and Clinical Language Sciences, University of Reading, Reading, United Kingdom; ^2^IIT, Genoa, Italy; ^3^Experience Insight, Bracknell, United Kingdom; ^4^Research Institute, Kochi University of Technology, Kami, Japan

**Keywords:** neuroscience, physiology, consumer behavior, methodology, marketing

## Abstract

The current paper investigates the value and application of a range of physiological and neuroscientific techniques in applied marketing research and consumer science, highlighting new insights from research in social psychology and neuroscience. We review measures of sweat secretion, heart rate, facial muscle activity, eye movements, and electrical brain activity, using techniques including skin conductance, pupillometry, eyetracking, and magnetic brain imaging. For each measure, after a brief explanation of the underlying technique, we illustrate concepts and mechanisms that the measure allows researchers in marketing and consumer science to investigate, with a focus on consumer attitudes and behavior. By providing reviews on recent research that applied these methods in consumer science and relevant related fields, we also highlight methodological and theoretical strengths and limitations, with an emphasis on ecological validity. We argue that the inclusion of physiological and neuroscientific techniques can advance consumer research by providing insights into the often unconscious mechanisms underlying consumer behavior. Therefore, such technologies can help researchers and marketing practitioners understand the mechanisms of consumer behavior and improve predictions of consumer behavior.

## Introduction

One of the goals of consumer scientists and marketing practitioners is to identify, predict, and understand the behavior of consumers. Self-report and behavioral measures have been widely used in consumer and marketing research to measure what consumers experience, think, and feel. Examples include self-report questionnaires, self-assessment manikins (SAMs) or other forms of behavioral data like reaction times in judgment and decision-making paradigms (see Poels and Dewitte, 2006, for a review). Research in consumer science has also employed physiological measures since the 1960s (Krugman, 1965; Plummer, 1972; Green and Tull, 1978; Klebba, 1985; Poels and Dewitte, 2006; Wang and Minor, 2008 for an overview of the history of this research). For example, early research in the 1960s used measures of pupil dilation to predict sales rates of products and effectiveness of advertisements (Krugman, 1965, but see Goldwater, 1972; Stewart and Furse, 1982; Wang and Minor, 2008). More recently, research in consumer science has implemented neuroscience-based techniques and identified the brain mechanisms underlying consumer behavior and decision making (Yoon et al., 2006; Lee et al., 2007; Hubert and Kenning, 2008; Kable, 2011; Plassmann et al., 2011, 2015; Camerer and Yoon, 2015; Hsu and Yoon, 2015; Genevsky et al., 2017; Hsu, 2017; Karmarkar and Plassmann, 2017 for an extended discussion of this issue). Indeed, neuroimaging using Positron emission tomography (PET) has been patented as a marketing tool by Zaltman and Kosslyn (2000), although fMRI has now largely superceded PET as a neuroimaging technique in marketing research.

The successful implementation of physiological and neuroscientific measures in the field has attracted much attention of practitioners and there are a number of companies that offer analyses of consumers’ physiological and/or neural responses to stimuli (see Hsu, 2017, for a review). In fact, physiological and neuroscientific techniques not only allow researchers to identify the mechanisms underlying consumers’ behavior. They can also reveal responses or reactions that consumers cannot or do not want to divulge, such as attitudes toward products or brands that are socially undesirable, that consumers are not consciously aware (e.g., Galdi et al., 2008; Ohme et al., 2009), or that they are not able to verbalize, such as when testing specific groups of consumers like children. This way, these measures might be able to permit better identification of consumer’s preferences and better predictions of their behavior than measures that simply rely on self-observation (Galdi et al., 2008; Plassmann et al., 2015).

The current paper aims to highlight how physiological and neuroscientific measures can be applied to complement research in consumer and marketing science. Drawing from recent studies in the field and current insights from research in relevant related fields, we aim to provide an overview of the application of the most prominent physiological and neuroscientific methods (see Smidts et al., 2014; Camerer and Yoon, 2015; Hsu, 2017; Karmarkar and Plassmann, 2017, for a review of the theoretical value of physiological and neuroscientific measures), covering the electrodermal response, heart rate, facial muscle activity, pupillometry, eye tracking, electrical brain activity, near-infrared spectroscopy (NIRS), and magnetic resonance imaging (MRI). We briefly introduce each method and highlight examples of its application in previous research from consumer science and relevant related fields for the measure. We focus on the ecological validity of these measures. Traditional research on these measures concerns physiological and neural responses to controlled laboratory tasks, raising the question as to whether they are useful for predicting or explaining behavior beyond the laboratory; and whether they can be used to measure consumers’ experience in real life. In this paper, we highlight recent findings that suggest such measures can indeed predict consumers’ emotional and affective response to products and brands, and most importantly actual behavior such as choice. We also discuss recent technical advances that have allowed the development of a number of wearable nanotechnologies that may circumvent the need for expensive laboratory based studies, or extrapolation of data to a larger population, by making it possible to collect large scale ‘big’ data in natural consumer environments.

## Physiological and Neuroscientific Measures Used in Consumer Research

### Electrodermal Activity (EDA)

Differences in the electrical conductance of the skin are measured under the umbrella term of electrodermal activity (EDA; Braithwaite et al., 2013), which is also known as the skin conductance response (SCR), or more traditionally as galvanic skin response (GSR); on which research dates back as far as the end of the 19^th^ century (Vigouroux, 1879; Neumann and Blanton, 1970; Fowles et al., 1981; Boucsein, 2012). These changes in skin conductance are a result of sweat emitted by eccrine glands. These glands cover the whole body, but the soles of the feet and palms of the hands have a denser coverage (Dawson et al., 2007). Skin conductance is recorded by attaching gelled sensors to the palmar skin and applying a small direct current (Fowles et al., 1981). Measurements of EDA include both background tonic [skin conductance level (SCL)] and rapid phasic components (SCRs) that can be measured in response to single presentations of stimuli.

Electrodermal activity is often considered as one of the most useful indices of sympathetic arousal, because in comparison to other measurements such as heart rate or pupil dilation it is not contaminated by parasympathetic activity (Figner and Murphy, 2011). Sympathetic arousal is a psychological state of heightened alertness that manifests ‘fight or flight’ changes to physiological processes including sweat production, pupil diameter or respiratory rate. Sympathetic arousal occurs as an autonomic response to a stimulus and is controlled by the sympathetic nervous system (conversely, the parasympathetic nervous system controls physiological processes when the body is at rest), so may be considered outside of the control of the individual. EDA has been widely used as an index of arousal reactions induced by emotionally evocative stimuli (Kreibig, 2010), outcomes of one’s choices, i.e., gains and losses (Bechara et al., 1999), anticipations of risks (Bechara et al., 1999, 2005; Critchley et al., 2001), and task difficulty (Pecchinenda, 1996; Krosch et al., 2012). In sum, measuring EDA is useful to assess consumers’ reactions and predict their decisions as well as to investigate the role of arousal in understanding how consumers arrive at and will respond to their decisions. Importantly, measurements of EDA can reveal these responses even when the consumer is not aware of them as described below.

Electrodermal activity has been widely used in consumer research (Shapiro and MacInnis, 2002; Bolls et al., 2003; Boucsein, 2012). For example, EDA has shown particular sensitivity to very subtle but effective changes in advertising stimuli. In one study (Ohme et al., 2009), EDA data were measured while participants viewed two highly similar television advertisements for the same skincare product. One advertisement contained an additional subtle hand gesture by a female model, while the other advertisement had the same information except for the hand gesture. Importantly, results from a prior study (Ohme and Pyl, 2006) showed that the version with the hand gesture elicited a greater number of product selections in a behavioral shelf test. The advertisement with the hand gesture was observed to elicit higher EDA than the other version, although participants reported no conscious awareness of the difference between the two advertisements. Thus, this study highlights that arousal contributes to the effectiveness of advertising, and therefore physiological measures on arousal, such as EDA, may be able to provide more precise measures about consumers’ choice than self-report measures. Note that enhanced arousal can arise from various external stimuli that consumers may encounter, such as store atmosphere (Crowley, 1993; Groeppel-Klein, 2005), and thus EDA might be useful to understand the effects of arousal on consumer behavior in various contexts.

Evidence for the effectiveness of EDA in the prediction of consumer preference is also provided by Guerreiro et al. (2015), who examined conditions under which arousal predicted consumer choice by recording EDA whilst displaying images of hedonistic products such as chocolate truffles, or utilitarian products such as washing detergent. They found that a higher increase in SCR, but also a shorter time to reach SCR peak, were significantly associated with consumer choice for hedonistic products. Conversely, for utilitarian products longer times to reach the arousal peak were more strongly associated with consumer choice. More recently, Vila-López and Küster-Boluda (2018) measured EDA and self-report ratings of perceptual opinion for different styles of product packaging before selecting their product of choice. For the most popular style, both EDA and self-report significantly predicted consumer outcome, however, for all other styles only EDA significantly predicted selection of the item. The study further highlights the advantage of physiological measures over self-report in the immediate prediction of consumer behavior. SCR has also been shown to be a reliable indicator of consumer choice over time. For example, Ohira and Hirao (2015) presented images and product descriptions for two cosmetic products whilst recording SCR, and participants were asked to evaluate the data and rate the products. Encountering the preferred product (as indicated by self-report) evoked a higher SCR. In addition, when the experiment was repeated with the same participants 1 year later, their preferences of products and corresponding SCR patterns remained stable, suggesting that SCR can be a predictor of consumers’ choice even 1 year later.

All of the studies presented above highlight that EDA is a reliable indicator of preference without the need for self-report, which may be advantageous when products may be of a sensitive or embarrassing nature, or where demand characteristics may influence subject responses. However, it is important to note that EDA is dependent on, and reacts to, many factors such as the room temperature and humidity, caffeine, circadian rhythm and time of day, medication, nicotine, or prior physical exercise, and using traditional equipment participants have to stay quite still during the measurement which can limit the nature and length of an experimental session (cf. Figner and Murphy, 2011). As such the technique may have limited application in consumer environments. However, wearable technology has shown reliable recording of EDA using wrist bands that may be worn in naturalistic settings, storing, or transmitting real-time data (Fletcher et al., 2010). Indeed Tschacher et al. (2012) demonstrated that EDA recordings, measured using a wireless glove while participants visited an art gallery, were significantly related to participants’ aesthetic-emotional experience. An additional point to note is that when it comes to emotional reactions, arousal is merely one of the dimensions of emotions. Emotional experiences differ in valence, i.e., they can be positive or negative (Russell, 1980; Russell and Carroll, 1999; Picard et al., 2016). While EDA is an effective measure for the capture of arousal, it is difficult to detect the valence of emotion induced by products when positive and negative emotions are not different in their arousal levels. Therefore, the technique is best used in conjunction with other methods more sensitive to valence of emotion such as facial expression analysis.

### Facial Expression Analysis (Determined by Video Capture) and Facial Muscle Activity [Determined by Electromyography (EMG)]

When researchers are interested in valence, the measurement of facial expression provides valuable insights. Facial expression analysis uses a video camera to capture facial images. These images are then coded manually by a researcher or automatically using computer software. Typically, all facial features within an image are coded (e.g., Ahn et al., 2010; De la Torre and Cohn, 2011). Conversely, in EMG analysis, there are only two muscle groups of particular interest: the corrugator supercilii of the eyebrows is associated with frowning and the zygomaticus major of the cheeks is associated with smiling (Larsen et al., 2003). By placing electrodes near these muscle groups, EMG is used to record the electrical impulses generated by the contracting muscles. These changes can even be detected when changes in facial expressions are not visually apparent (Cacioppo et al., 1986), providing implicit measurements of positive and negative affect. This suggests that EMG may be more sensitive than facial expression analysis; however the technique is more invasive and, unlike video capture, cannot be carried out covertly.

In consumer science, facial expression analysis has been shown to be effective in evaluating consumer opinions about products (Lajante et al., 2017). For example, Danner et al. (2014) videoed the expressions of young adults as they tasted different orange juice samples. Images were recorded overtly and participants were asked to explicitly make a facial expression that reflected their opinion of the product. Under these conditions, image analysis was able to discriminate between the facial reactions following each of the different juice samples. Further, predictions of liking and disliking of the products determined through analysis of the facial reactions correlated well with self-report ratings collected at the same time. Of particular interest, the authors also repeated the experiment with covert videoing of participants and no prompting of facial expressions. The technique was similarly effective at recognizing negative emotional responses to the juices, although it was not effective for positive emotional responses, possibly due to the subtlety of many natural, positive facial expressions.

Facial EMG has similarly been used to assess valence of affect toward products and advertisements (Hazlett and Hazlett, 1999; Poels and Dewitte, 2006). For example, radio adverts with a positive tone evoked more zygomatic (‘smiling’ muscle) activity and those with a negative tone more corrugator (‘frowning’ muscle) activity (Bolls et al., 2001). EMG also appears to be a sensitive indicator of processing fluency (Winkielman and Cacioppo, 2001; Schwarz, 2004) which is another source of positive valence (Reber et al., 1998; Schwarz, 2004). Fluency reflects the ease with which information can be perceived and processed (Alter and Oppenheimer, 2009). Further, fluency increases interpersonal trust (Zürn and Topolinski, 2017). Winkielman and Cacioppo (2001) recorded facial EMG whilst presenting images of everyday objects to their participants for evaluation, while manipulating their processing fluency by extending stimuli viewing times or by presenting contour primes immediately prior to each stimulus. In accordance with previous findings (e.g., Schwarz, 2004), participants expressed greater preference for products with higher fluency than those with lower fluency. In addition, Winkielman and Cacioppo (2001) found that activity of the zygomaticus smiling muscles was greater when fluency was enhanced.

An alternative application of EMG involves the registering of eye-blinks. Eye-blinks occur as part of the startle reflex (Graham, 1975) which can be modulated by valence/pleasantness (Vrana et al., 1988): a startle reflex toward a burst of white noise following the presentation of a positive stimulus was observed to be smaller in magnitude than one following the same burst of noise after an unpleasant stimulus (Lang et al., 1998). A recent marketing study applied this principle to brand attitude (Walla et al., 2011). Brand names, which had previously been rated on liking, were presented to participants. Each presentation was followed by a burst of white noise, to evoke a startle reflex which was measured by EMG. The least liked brands evoked greater eye blink response amplitudes than the preferred brands. These findings indicate that recording eye blinks with EMG provides a sensitive measure of brand attitude and confirm previous EMG findings in facial muscle activity responses to affective images (Lang et al., 1990).

In short, facial expression analyses and EMG are useful to measure positive and negative affect toward products and advertisements. These techniques can be used to investigate the role of preferences, but also of brand attitude, in the consumer decision making process. However, there are also several methodological limitations or challenges. The facial expression analysis requires a clear, well lit, direct facial image to be recorded. Changes in head position or lighting, for example, make it difficult for automatic software to accurately map facial features (although technological advances are continually being made). Automated coding of images also requires the creation of large reference databases of images. However, the main advantage, compared with the other techniques described here, is that data may be collected covertly.

In EMG, the sensors may pick up not just affective responses of facial muscles, but also confounding activity such as speech and (mental) fatigue-related activity (van Boxtel, 2010). The participants’ responses could be also modulated by demand characteristics, as they are likely to be aware of the nature of the experiment and what the EMG electrodes record (Bolls et al., 2001; Poels and Dewitte, 2006); EMG cannot discriminate a real or fake smile. Furthermore, measurement accuracy is dependent on precise sensor placement (Chen et al., 2004). In addition, the sensors may also pick up body movements as well as the movement of facial muscles (Bolls et al., 2001). However, as in EDA, recent developments suggest that wireless, non-invasive devices can be used for measuring facial muscle activity even when consumers are moving freely (Inzelberg et al., 2018). Currently such devices allow the precise measurement of strong facial muscle activity only, but are comfortable and unobtrusive enough to be worn for long periods of time.

### Heart Rate

Heart rate has been shown to be effective as a real-time measure of arousal, attention, cognitive effort, or physical effort (Bolls et al., 2003; Poels and Dewitte, 2006; Sung et al., 2016). Deceleration of heart rate has been associated with increased activity of the parasympathetic nerve system (PNS), whereas acceleration of heart rate has been associated with increased activity of SNS (e.g., under increased arousal; Acharya et al., 2006). In addition, people typically show prolonged cardiac deceleration during perception of novel or salient stimuli (e.g., Bradley et al., 2001), which has been associated with stronger orienting reactions to and enhanced attention/perceptual processing of the stimuli (Lang, 1990; Bradley, 2009). Arousal and attention are both important factors in determining the effectiveness of advertising stimuli and are interlinked (Lang, 1990; Bolls et al., 2001). Therefore, heart rate may be a useful measure to predict the effectiveness of advertisements.

A study investigating physiological responses toward popular brand logos versus advertising for disliked brands (Maxian et al., 2013) recorded heart rate, SCR, and facial muscle activity. Well-loved brand logos (based on a global scoreboard) elicited greater arousal levels as measured by self-report ratings. Importantly, these brands evoked a prolonged heart rate deceleration and greater ‘smiling muscle’ EMG activity (Maxian et al., 2013), indicative of stronger attention allocation to the brands and stronger positive affect, respectively. In contrast, the SCR data showed no significant change in arousal in response to the popular brands. These findings further highlight the added advantage of various physiological measures when combined with participants’ self-report.

In another study by Sung et al. (2016), participants were presented with an advertisement with or without the word ‘new’ in a salient position. It was found that the presence of the word ‘new’ increased the viewing time for advertisements. This had no effect on self-reported liking of each advertisement; however, a corresponding heart deceleration was observed for the advertisement with the word ‘new,’ again highlighting the value of heart rate in elucidating the effects of advertising strategies (see also Bolls et al., 2001; Lang et al., 2002). Combined, recent studies provide evidence for the sensitivity and versatility of heart rate measurement to even subtle aspects of information presented to consumers.

In addition, while research in consumer science and marketing research has mostly focused on heart rate, previous research has highlighted the value of investigating heart rate variability (HRV) which refers to variation in the time interval between heartbeats based on measurements of at least a few minutes (Sinnreich et al., 1998; Acharya et al., 2006; Shaffer et al., 2014). Higher HRV is associated not only with better physical fitness, but also with improved levels of self-regulation, such as the successful regulation of emotion, more positive emotions, or enhanced sensitivity to social feedback, and to one’s ability to predict others’ emotions accurately and to feelings of social connectivity (Thayer and Lane, 2000; Porges, 2001; Kok and Fredrickson, 2010; Muhtadie et al., 2015; Sakaki et al., 2016). In contrast, lowered HRV has been linked to stress, anxiety, and exertion (Shaffer et al., 2014). HRV may thus be useful in order to identify the underlying mechanisms of (mal)adaptive emotion and self-regulation processes in consumer behavior and to distinguish different groups of consumers.

The heart rate studies described above are all laboratory based; however, with the development of new technologies such as smart watches, it is now possible to measure HR and HRV in naturalistic consumer settings. An increasing number of studies have documented the reliability and validity of these tools (e.g., Wallén et al., 2012; Heathers, 2013; Liu et al., 2013; Akintola et al., 2016). For example, researchers measured participants’ heart rate for 3 weeks with a wearable heart rate monitor and found that individuals’ HRV was affected by the degree of social support they received at that time (Gerteis and Schwerdtfeger, 2016). These results are consistent with earlier laboratory-based studies that observed HRV to be associated with stress (e.g., Shaffer et al., 2014) and suggest that measuring physiological responses to real-life events/stimuli is a promising way to estimate consumers’ experience in real life. However, it should be noted that it is also important to take into account age, physical fitness level, or medication when comparing heart rates between participants.

### Pupillometry

Another technique related to arousal is pupillometry; a technique to measure changes in pupil size (Sirois and Brisson, 2014; Mathot, 2018). Pupil dilation and constriction occur in response to changing light conditions, luminance and brightness (Bradley et al., 2008; Venkatraman et al., 2015), but variations in pupil size also reflect the intensity of mental activity and responses to emotional stimuli (Laeng et al., 2012; Sirois and Brisson, 2014). Seminal research in the 1960s has shown that the pupil dilates when participants experience increased cognitive processing demands such as when performing mental arithmetic (Hess and Polt, 1960) or when remembering complex information (Kahneman and Beatty, 1966; see Goldwater, 1972, for a review). Early research also suggested that pupils dilate in response to pleasant stimuli and contract when viewing unpleasant stimuli (Hess, 1968; see review by Goldwater, 1972). Pupil dilation has subsequently been considered as a measure of preference which was further supported by evidence that greater pupillary increases for products or advertisements correlated with sales rates and the effectiveness of the advertisements (Krugman, 1965). However, the reliability of this research has been questioned (Goldwater, 1972; Stewart and Furse, 1982) and it is unclear whether the correlations are due to pupil dilation reflecting liking of products and advertisements or rather increased interest, in-depth processing, or arousal.

More recent research has confirmed that the pupil dilates in response to emotionally arousing materials but this effect appears to be independent of valence. In a seminal study, Bradley et al. (2008, 2017) presented participants with positive, negative, and neutral images. Positive and negative images were matched for arousal level. Bradley and colleagues observed greater pupillary increases for both pleasant and unpleasant images in comparison to neutral images indicating that pupil dilation is an indicator of emotional arousal, and not hedonic liking (but see Ho and Lu, 2014). Further, pupil dilation is associated with the activity of the locus coeruleus (Murphy et al., 2014) which is a key region of emotional arousal irrespective of valence. Thus, it appears that pupil dilation reflects activity of the SNS induced by presentation of emotional stimuli and is indicative of intense emotional arousal toward both pleasant and unpleasant stimuli and experiences.

In consumer science, various studies have reported increases in pupil size in response to stimuli and experiences that evoke strong interest and emotions in consumers, such as when consumers listen to music that they experience as enjoyable and emotion-evoking (Laeng et al., 2016) or when viewing human faces (Blackburn and Schirillo, 2012) or art that are experienced as aesthetically pleasant (Johnson et al., 2010). Increases in pupil size also allow researchers to differentiate consumers based on their interests, for instance, pupillary increases in response to (erotic) images of female and male models correspond with the sexual orientation of observers (Rieger and Savin-Williams, 2012; Attard-Johnson and Bindemann, 2017; Watts et al., 2017) and impulsive, but not non-impulsive, buyers display enhanced pupil dilation when they are presented with shopping scenes (Serfas et al., 2014). Pupillary measures are thus useful to measure whether products, experiences or people evoke interest and emotional reactions and arousal in consumers.

Measuring pupillary changes can also help to uncover the processes underlying decision making and responses to reward. For instance, pupil dilation has been related to the experience of cognitive surprise when encountering conflicting or unexpected information (van Steenbergen and Band, 2013; Braem et al., 2015). Specifically, participants have demonstrated changes in pupil size if the outcome of a choice did not match their expectation (Preuschoff et al., 2011; Braem et al., 2015). Mismatch of expectation can influence subsequent decision making and liking of products and sellers (cf. Gneezy and Epley, 2014). Measuring pupillary responses toward potentially surprising information would thus allow researchers to investigate whether an event evokes such cognitive surprise in a consumer and whether and how it subsequently impacts the consumer’s behavior and decisions. Further, in difficult decision-making circumstances, pupil dilation appears to index the processes involved in decision making such as raising decision thresholds when presented with two positive ‘win’ choices (Cavanagh et al., 2014) or the exploration of changing reward contingencies (Jepma and Nieuwenhuis, 2011). Finally, Bijleveld et al. (2009) found increased pupil sizes in response to reward that required the observer to invest mental effort.

In sum, pupillary responses appear to indicate emotional arousal, increased mental activity in decision making and in response to reward. Despite some evidence that people are able to control their pupil size voluntarily (Ekman et al., 2008) this control may only be indirectly achieved by imagining events that would normally evoke pupil dilation (Laeng et al., 2012). Additionally, it is difficult to suppress pupillary responses at will (Laeng and Sulutvedt, 2014). The fact that people have little influence over their pupillary response, and that this response occurs in the absence of voluntary processes (Laeng et al., 2012), gives pupillometry an advantage over many other measures such as self-report. Although the above studies were all laboratory based, the advent of wearable technology means that portable systems may now be used to measure pupil changes in consumer-based locations, potentially increasing the number of marketing applications for this technique. However to-date, no such studies were evident in the literature.

### Eye Movements

Eye tracking equipment can be used to measure changes in pupil size, as described in the previous section, but it is also used to monitor the direction of gaze in consumer research (see Wedel and Pieters, 2007, for a review). Modern eye trackers use specialized sensors to infer viewing direction from the patterns of infrared light reflected by the cornea during normal eye movements. These sensors can be placed on a table top, or in a pair of specialized goggles to allow mobile eye tracking outside of the laboratory.

The underlying assumption of eye tracking is that an individual is visually and mentally processing any stimuli toward which their gaze is directed. Eye tracking thus represents a good measure of visual attention (Wedel and Pieters, 2007; Atalay et al., 2012). Importantly, eye tracking is useful when aiming to investigate whether and how attention is allocated to stimuli, for instance whether they capture attention or whether attention dwells on them. Measuring eye movements is also helpful in understanding the role of vision in judgment and decision making, motivation and goal pursuit, and preferences (cf. Russo and Leclerc, 1994; Orquin and Mueller-Loose, 2013). Indeed, eye movements have been linked with preference formation using an evidence accumulation framework [attentional drift diffusion model (aDDM); e.g., Krajbich et al., 2010, 2012; Krajbich and Rangel, 2011].

Eye movement data are categorized into two types: fixations and saccades (Zurawicki, 2010). A fixation is the duration for which the eyes remain continuously directed at a specific location. Analysis of fixations can be used to examine whether attentional resources are preferentially allocated to a particular stimulus over other stimuli and is often also referred to as dwell time. A saccade records the speed and angle of an eye movement from one location to another and can be used as an indicator of how quickly a stimulus grabs attention. In addition to measuring attentional capture and maintenance by certain stimuli, eye tracking data also reveal when information causes avoidance or inhibition of attention. Further, eye tracking, in contrast to cognitive measures of attention allocation (see Yiend, 2010, for an overview), is well-suited to identify the time course of attention allocation (McSorley et al., 2017).

Studies using eye tracking have delivered important insights into how consumers process and attend to marketing materials such as advertisements or catalogs and how such attentional patterns relate to choice and consumer behavior. In particular, research with eye tracking has allowed the identification of aspects of a design that automatically grab attention because they are salient (cf. van Zoest et al., 2004; see Wolfe and Horowitz, 2017, for an overview of salient features guiding attention). For instance, the usefulness of eye tracking as a research tool was highlighted by Lohse (1997; see also Lohse and Wu, 2001). In his study, participants were asked to select a company from the yellow pages based on a specific assignment (e.g., “buying flowers for a friend”). Eye tracking data revealed that participants noticed the majority of large adverts but only a quarter of the plain listings. Color adverts were viewed more often and for longer than black and white adverts (but see García-Madariaga et al., 2018). Moreover, the advertisements that were eventually selected were viewed for a longer duration relative to other advertisements, which suggests that directed attention is a predictor of consumer choice.

In a related vein, Janiszewski (1998) investigated the impact of display characteristics and catalog lay-outs during an exploratory search task. In the study, participants were asked to browse through a booklet depicting catalog pages. Results revealed a greater number of eye fixations on products with less surrounding competition from other products. Additionally, participants showed greater memory recall for products with longer fixation durations. Other research has shown that centrally presented information is more attention grabbing than information presented in the periphery (Atalay et al., 2012), though this can also result in enhanced attentional avoidance of branding activity when such information is displayed too long (Teixeira et al., 2010). Eye tracking research has also revealed how the composition of marketing information guides attention and subsequently impacts evaluation and choice. For instance, in a study by Palcu et al. (2017) participants were presented with online advertisement banners showing faces and products. The authors manipulated whether the models’ gaze was directed at the product. Interestingly, when the face gazed toward the region where the product was presented participants’ likelihood of looking at the advertised product increased and buying intentions toward the product were higher.

It is important to realize though that attention allocation varies with the activated goal (Pieters and Wedel, 2004). For instance, recent research has shown that salient stimuli such as highly emotional events will not grab attention when they are not goal relevant (Vogt et al., 2013; see also Lee et al., 2014). Additionally, a recent study by Townsend and Kahn (2013) highlighted that when participants are presented with large choice sets they are more likely to scan all options thoroughly when options are verbally rather than visually presented, presumably because visual information activates a more holistic and superficial processing mode whereas verbal information prompts consumers to process in a more detailed way. Further, Teixeira et al. (2012) highlighted that positive emotions and especially surprise concentrate attention on online advertisements. In sum, this research shows that eye tracking allows researchers to understand which features of marketing materials and product information are salient and thus grab consumers’ attention but it can also help to understand how consumers process information, and under which conditions all available information is used.

Eye tracking can also be implemented to study the processes underlying various relevant effects in consumer science such as consumers’ preferences, judgment and decision making, or goal priming. For instance, a recent study by van der Laan et al. (2017) investigated how the priming of health-related motivations impacts consumers’ food choices. In this study, participants chose between high and low energy food products in a realistic online supermarket task while their eye movements were recorded with an eye-tracker. van der Laan and colleagues found that the health goal prime caused an increase in choice of healthy low energy food choices and a decrease in unhealthy high energy food choices in comparison to two control groups. Importantly, this effect was mediated by enhanced dwell time on low energy food items. In a related vein, Büttner et al. (2014) found that highly impulsive buyers display an attentional bias toward products presented as distractors in a shopping scenario. This was indicated by enhanced dwell time toward distractor products and reduced dwell time on focal items. This research thus suggests that at least one mechanism that mediates the effect of currently activated motivations and goals, and motivational predispositions on consumer behavior is the tuning of attention toward relevant information (cf. Cian et al., 2014; Vogt et al., 2017). In rapid decision making, product salience has also been shown to play an important role (Milosavljevic et al., 2012). Further, eye tracking has revealed that attention underlies or indexes consumers’ preferences and decisions. For instance, eye tracking can predict preferences prior to conscious decision making (Shimojo et al., 2003; see also Muñoz-Leiva et al., 2018); when shown a pair of faces, participants’ gaze gradually shifted toward the face they eventually rated as more attractive. Interestingly, when gaze direction was manipulated, decisions were biased in favor of the direction of forced gaze (cf. Shen and Rao, 2016). Similarly, Cavanagh et al. (2014) found that eye movement predicts decisions (whereas pupil dilation predicts shifting decision thresholds); it further predicts the interpretation of ambiguous material (Everaert and Koster, 2015).

Modern eye trackers are precise and can sample the gaze location 1,000 times per second with 0.1° accuracy, and as with pupillometry, the technology can now be contained within wearable technology that may be used to investigate consumer attention in naturalistic environments. For example, Babcock et al. (2002) used portable eye trackers to investigate attentional gaze when taking, cropping, and viewing digital photographs, in order to understand customer dissatisfaction with digital printing services. However, there are some technical limitations to eye tracking; available technology cannot track the direction of gaze during blinking and does not work for all participants, for example those with glasses, heavy make-up, dark eyelashes, or excessive tear fluid (Pieters et al., 1999). Moreover, there are additional challenges in using eye tracking to measure ‘attention,’ which is a broad and complex process (see Luck and Vecera, 2002, for an overview). Specifically, eye tracking may not be useful in investigating *covert* attention; the (re-)orienting of focus without moving one’s eyes (Posner, 2008). In such circumstances, attention may be shifted, but there is no detected fixation or saccade present (Armstrong and Olatunji, 2012). Importantly covert attention is mainly relevant when studying early attention such as when studying attention in the first 200 ms of encountering a stimulus.

### Electroencephalography (EEG)

When neurons in the brain are active or ‘firing,’ they emit a small electric current. Using sensors placed on the human scalp, electroencephalography (EEG) can pick up these signals (Berger, 1929; Luck, 2014). Analysis of the location and strength of these ‘brainwaves’ can provide information about the types of mental processes that are occurring. An alternative approach, magneto-encephalography (MEG), detects the magnetic field generated by the same neuronal activations and is thus based on the same principles as EEG. The temporal resolution of EEG is high, meaning that changes in brain activity can be detected milliseconds after they occur. However, spatial resolution of EEG is limited, meaning that the exact source of specific brain activation may often be difficult to pinpoint. It is still possible, however, to identify the general source of EEG signals (e.g., left-sided, right-sided, and central activation at occipital, parietal, temporal, central, and frontal areas).

Electroencephalography techniques can be further classified into two sub groups, either employing the detection of brain-wave oscillations or detecting event-related potentials (ERPs). Brain-wave oscillations are reflected by rhythmic activity in the EEG signal when groups of neurons synchronize their firing patterns. These oscillations are generally classified into frequency bands, which are thought to reflect differential processes depending on their location (Venkatraman et al., 2015). For example, alpha waves oscillate between 8 and 12 Hz (Berger, 1929) and can be detected on the posterior scalp when people are asleep or relaxed. Evidence suggests that alpha power may be inversely proportional to the level of cortical activity occurring in the underlying brain regions (Coan and Allen, 2004). In response to stimuli, variations in alpha activity have been observed at different scalp positions, which may allow the formation of inferences about the processing of the stimuli. For example, asymmetry of alpha activity in the frontal brain areas can be used as a measure of affect toward a stimulus, as greater alpha activity on the left side, compared with the right, has been previously observed to be an indicator of positive affect and associated with the approach motivation. Conversely, greater relative alpha activity on the right side has been observed to be an indicator of negative affect and linked with the avoidance motivation (Davidson et al., 1990; Davidson, 2000, 2004; Harmon-Jones et al., 2010).

Event-related potentials are direct neural responses to sensory or cognitive events (Luck, 2014). When regional brain activity is time locked to a stimulus presentation, the neuronal activation immediately following each presentation can be averaged across multiple trials, plotting a characteristic wave. The various components of the ERP wave have been implicated in specific cognitive and affective processing (Sutton et al., 1965; Luck, 2014). One example waveform component is the P300, named because it has a positive peak that occurs approximately 300 ms after stimulus onset. This distinctive waveform was discovered by Sutton et al. (1965) who found this peak to be larger when participants could not predict whether the next stimulus would be visual or auditory. Later research discovered that the P300 reflects attentional capture by positively affective stimuli (Palomba et al., 1997; Cuthbert et al., 2000; Hajcak et al., 2010). Another distinctive waveform example is the N200: a negative potential occurring approximately 200 ms after stimulus onset, and primarily detected over anterior scalp sites (Folstein and Van Petten, 2008). This component has been related to mismatch negativity; the response is elicited by an infrequent stimulus among a stream of frequent stimuli (Patel and Azzam, 2005; Folstein and Van Petten, 2008). Under conditions where the visual characteristics of a stimulus ‘pop out’ and are easily identifiable, the N200 may be detected in posterior locations (Patel and Azzam, 2005). Measuring ERPs, therefore, rapid responses to a stimulus can be determined with great timing precision, and the spatial location of specific waveforms provide information about the nature of the underlying cognitive processes. Since EEG allows us to measure various mental processes it can be informative at all stages of the consumer decision process, such as mismatch detection (Garrido et al., 2009), valence determination (Davidson, 2004), and memory encoding and retrieval (Klimesch, 1999; Weiss and Rappelsberger, 2000).

In consumer science, both ERPs and oscillation frequencies have been found to be associated with product choice (see Lin et al., 2018). For example, Telpaz et al. (2015) showed participants a series of common household product images whilst recording theta oscillations (5–8 Hz; related to inhibition of response) and the N200 ERP component on a mid frontal site (related to choice). The EEG session was followed by a selection task, during which participants were shown pairs of two products and asked to choose one of them. Products that the participants subsequently chose in this selection task elicited weaker theta oscillation and greater N200 responses during the EEG session than those that the participants subsequently did not choose. These findings suggest that different cognitive processes occurring during evaluation are reflective of product preferences, which may later predict the outcome of a purchase decision.

Other ERP waveform components, similarly thought to reflect preference, were investigated following passive presentation of luxury brand images and basic brand images with or without a viewing companion (Pozharliev et al., 2015). Waveform components analyzed in this study were the P200, which has been implicated in emotional arousal (Amrhein et al., 2004; Olofsson et al., 2008); the P300, which is often evoked by attention capture for positively affective stimuli (Palomba et al., 1997; Cuthbert et al., 2000; Hajcak et al., 2010); and the late positive potential (LPP), which is observed for pleasant and unpleasant stimuli compared with neutral stimuli (Olofsson et al., 2008) and implicated in sustained attention toward affective stimuli (Lang and Bradley, 2010). Regardless of luxury or basic brand status, product preference as indicated by the P200 and P300 components was larger when participants were with a companion rather than being alone. Interestingly, the LPP was larger for luxury products than for basic products, but again only in the companion condition. This study demonstrates that laboratory based EEG techniques are sufficiently sensitive to determine effects such as the enhancement of positive evaluation in the presence of others, or to use the real world parallel, when shopping with friends.

Another study by Khushaba et al. (2013) used EEG to investigate preferences for various cracker features: shape, flavor, and topping. Phase synchronizations in delta, theta, alpha, beta and gamma bands, between left and right frontal and occipital locations, were indicative of cognitive processing during the decision process. Importantly, modulations of theta band activity across left occipital regions allowed discrimination of the features that were most important to the consumer; in this case flavor and topping were found to be more relevant to preference than shape. Therefore, EEG can be effectively used in discriminating the importance of different features during product evaluation.

Above, we commented on the poor spatial resolution of EEG. However, using dense arrays with a high number of electrodes, the spatial resolution can be increased (although spatial resolution in EEG is still lower than that of MRI). In a recent study by Daugherty et al. (2016), participants first watched short direct response style television advertisements (where viewers are prompted to buy the product immediately via telephone or online). Brain activity was recorded using dense-array EEG equipment whilst they were shown photo-stills taken from the advertisements. Some of the adverts were known to have led to market success while others had been unsuccessful. It was found that, compared with unsuccessful advertisements, the successful ones were characterized by increased activity in the prefrontal cortex and pars triangularis; and reduced activity in the left anterior prefrontal cortex, right medial prefrontal cortex and posterior fronto-median cortex. The study highlights the improved spatial resolution of EEG when using dense arrays, and how this can provide valuable information about the processes underlying purchase decisions.

Although the use of traditional EEG arrays is restricted to the laboratory, the development of innovative new wearable technology means that EEG may soon be carried out in the field, further increasing the ecological validity of this technique (see Byrom et al., 2018 for a review of the latest innovations in wearable brain monitoring technology).

### Magnetic Resonance Imaging (MRI)

Functional magnetic resonance imaging (fMRI) allows us to look into the blood-oxygen-level dependent (BOLD) signals in the brain. This method is based on the assumption that when neurons in specific brain regions fire, they take up oxygenated hemoglobin, which is followed by an increase in oxygenated hemoglobin a few seconds later, which can be detected using MRI (Huettel et al., 2009). A key advantage of fMRI is its high spatial resolution (typically 2–3 mm^3^), meaning that active brain regions can be pinpointed with detailed accuracy, yet this non-invasive method also has an acceptable temporal resolution of about 2–5 s. Neuroscience research allows us to create a map of regions and brain networks that are associated with different mental processes. In consumer research, the technique can be applied in order to elucidate the underlying brain mechanisms of consumer behavior (Plassmann et al., 2011, 2015). fMRI has been used to study a range of mental processes, covering attention, arousal, affect, reward, decision making, and memory (Wang and Minor, 2008); processes that are highly relevant to consumer behavior. The use of fMRI in consumer research has been extensively reviewed (e.g., Plassmann et al., 2011, 2015; Solnais et al., 2013; Venkatraman et al., 2015; Cruz et al., 2016; Karmarkar and Plassmann, 2017). Here, we focus on fMRI studies that have targeted mental processes difficult to investigate using other physiological measures such as implicit processing and reward mechanisms.

Through the use of brain imaging, previous research has highlighted the importance of brain regions implicated in reward in consumers’ decision making. For example, Chang et al. (2016) showed participants images of clothing items modeled by celebrities, non-celebrities, or unworn with a written description and asked them to rate each image on its attractiveness and purchase intention. During ratings of product attractiveness, clothes worn by celebrities, relative to those worn by non-celebrities, evoked greater activation bilaterally in the lingual gyrus, which is associated with visual processing. In contrast, clothes worn by non-celebrities evoked greater activations in the left angular gyrus – a region which has been implicated in social and self-related cognitive processes (Bzdok et al., 2016), such as mental projection of one’s own body into pictures from an egocentric perspective (Ganesh et al., 2015). During ratings of purchase intention, non-celebrity worn garments produced greater activations in the caudate nucleus – a region implicated in reward (Nagai et al., 2016). Consistent with the enhanced caudate activity in the non-celebrity condition compared with the celebrity condition, participants also rated the garments in the non-celebrity condition most attractive.

In another study by Erk et al. (2002) participants were scanned while viewing images of cars and were asked to rate each car for attractiveness. Sports cars were rated more attractive than small cars or limousines and elicited greater activity in the right ventral striatum, left orbitofrontal cortex, left anterior cingulate, and bilateral prefrontal cortex. In a more recent study by Schaefer and Rotte (2007), participants viewed brand logos for car manufacturers while imagining driving a car of that model. Following the scanning procedure participants were further required to provide ratings of brand attractiveness, familiarity, whether the brand was a luxury or sports brand, and whether the brand would be a rational choice. An increased activity in the striatum was found for brands rated as luxury, but decreased striatum activity was observed for rational brands. Additionally, attractive brands engaged a network including the ventral striatum and the dorsolateral prefrontal cortex more strongly than did non-attractive brands. The areas of activation observed by both of the above studies are associated with reward and reinforcement; specifically the striatum has been identified as a so-called reward area (Schultz et al., 1997; Delgado et al., 2000; McClure et al., 2004) and the dorsolateral prefrontal cortex has a role in cognitive control (MacDonald et al., 2000) and working memory (Barch et al., 1997; Curtis and D’Esposito, 2003). These findings suggest that brand categorizations and preference for brands can be predicted by their associated reward value. Brain imaging is therefore a valuable tool in the investigation of these underlying reward mechanisms when consumers make product evaluations.

Previous research also suggests that fMRI is sensitive to consumer purchase decisions that may not be evident from self-report measures. In one study (Vezich et al., 2017), researchers investigated the marketing of environmentally friendly products after noting that self-reported preference ratings for ‘green’ products often did not translate into actual purchasing behavior. During the scanning procedure, participants viewed advertising posters for a range of environmentally friendly products and matched control products. Participants reported greater liking for the green products, however, fMRI results revealed greater activations in the ventral striatum and ventromedial prefrontal cortex whilst viewing the control adverts; areas associated with personal value and reward (O’Doherty et al., 2006; Hare et al., 2011). Furthermore, this activity showed a positive correlation with liking ratings for these standard products; the same relationship was not observed for the green products. The study highlights the ability of fMRI to provide an explanation for behaviors that do not match self-reported attitudes, for example, where self-report measures are biased by factors such as social desirability concerns.

Reward values for products may also be formed post-purchase; self-report measures such as consumer surveys suggest satisfied customers may maintain or adjust their post-purchase intentions favorably toward a purchased product (Tam, 2004; Kuo and Wu, 2012). fMRI research has provided physiological evidence for this in an investigation of attitudes to rival soda brands by McClure et al. (2004). McClure and colleagues observed that when participants were unaware of the brand they were drinking, reward related neural responses in the ventromedial prefrontal cortex correlated with the participants’ self-reported preferences. However, when participants were aware of the brand they were drinking their neural response showed heightened activity in the hippocampus and dorsolateral prefrontal cortex; areas implicated in recalling and modifying affective behavior, respectively. Therefore, fMRI is able to discriminate purely affective preferences from those biased by previous knowledge of brands.

### Near Infra-Red Spectroscopy (NIRS)

Functional near infra-red spectroscopy (fNIRS) utilizes NIRS to monitor functional changes in hemoglobin oxygenation in the cortex. Although slightly less spatially sensitive than fMRI, fNIRS results are directly comparable (Mehagnoul-Schipper et al., 2002). The technology is also less expensive than fMRI and is portable, potentially allowing cerebral blood flow changes to be observed in real-life consumer settings. Participants simply wear a light-weight head band over the forehead, containing an array of light sources and detectors. Indeed, NIRS is small and discreet enough to be incorporated in a hat, and advances in wireless technology mean that real-time transmission of data is already possible from the majority of consumer locations. Using fNIRS, Krampe et al. (2018) devised an experimental paradigm where participants viewed first-person video footage of supermarket aisles with and without merchandising communications. The study observed differential activity in the prefrontal cortex, specifically the orbitofrontal cortex and dorsolateral prefrontal cortex when comparing communication strategies, demonstrating the sensitivity of the technology for observing consumer neural activity. Çakir et al. (2018) devised a similar experiment where participants viewed a range of product images and were asked to indicate whether to buy or reject the item using a key press. In this case, significant differences in neural activity between bought and rejected items were observed in orbitofrontal regions, with greater activity observed for bought items. Again, this study demonstrates the sensitivity of fNIRS in detecting and distinguishing neural activity related to consumer decision making processes. Field trials where the technology is deployed in a real-world consumer setting have yet to be published; however, these initial investigative studies suggest that fNIRS is a promising technology for future application in consumer science.

### Combining Measures

While we have discussed each method separately, combining techniques may add to the pool of available data from which useful predictions can be made. For example, Venkatraman et al. (2015) measured eye movements, heart rate, EDA, BOLD, EEG, and self-report responses toward advertisements. They then examined whether these physiological and brain responses significantly predicted the market-level response to advertisements (i.e., sales and gross rating points). The results indicated that while traditional self-report measures had the strongest effect in accounting for variances in the market-level response, the BOLD signal from the ventral striatum significantly predicted the market-level response even after controlling for the effects of self-report measures; the stronger activity in the striatum while watching the ads was associated with better success of the ads at the market level. In addition to the BOLD responses, eye tracking and EEG measures also significantly predicted the market-level response when the effects of self-report measures were not controlled. Furthermore, other studies suggest that EEG can help predict future behavior even after controlling for self-report measures (e.g., Boksem and Smidts, 2015). These examples suggest that by including a range of complementary measures, a large proportion of variance in market level response can be accounted for. Therefore, it is useful to maintain a multi-method approach to study the forecasting of behavior. Technical advances are also being made in the study of the relationship between genes, molecular pathways, and consumer decision making behavior (see Smidts et al., 2014, for a discussion). Different techniques offer complementary information about association, necessity, or sufficiency in relationships between physiology, the brain, and consumer behavior (Kable, 2011; Hsu, 2017).

## Discussion

In the previous section, we have reviewed evidence showing that physiological and brain responses from a sample of participants are often associated with preference and choice in that same group. While these findings suggest that physiological and neuroimaging measures complement behavioral measures and help predict consumers’ behavior and choice, criticisms have been raised regarding the generalizabilty of findings, and the inference of causality in such studies (Plassmann et al., 2015; Mileti et al., 2016; Karmarkar and Plassmann, 2017).

### Generalizability

Previous studies have typically included a relatively small number of participants (approximately 25–40 in each condition), raising the question of whether the results can be generalized to another sample. We have described instances where new technologies may enable large scale data collection in naturalistic consumer environments. However, many neuroimaging and physiological devices are currently not portable and are also susceptible to artifact/noises, requiring researchers to incorporate many trials for a single condition to estimate reliable response. Due to such requirements, most previous studies tested participants’ responses in a controlled laboratory setting, raising further questions about whether the results obtained in these laboratory studies can be used to predict consumer’s behavior in real-life. Indeed, one of the important goals of applied consumer science and marketing research is to make an inference about behavior of the general population, beyond the sample collected within a single project.

Accumulated evidence indicates that physiological and brain measures recorded for a small group of participants complement self-report/behavioral measures in predicting people’s behavior in larger independent populations (Falk et al., 2012, 2016; Berkman and Falk, 2013; Genevsky and Knutson, 2015; Karmarkar and Yoon, 2016). For example, Genevsky et al. (2017) used fMRI and self-report questionnaires to determine participant preferences for existing crowdfunding projects. Participants were asked to view information about different projects whilst undergoing scanning. Participants indicated whether they would like to fund each project. After scanning, participants completed questionnaires to rate their liking, predictions of success, and level of arousal, in response to each project. Activations in both the nucleus accumbens and medial prefrontal cortex correlated with participants’ selection behavior, as did all self-report ratings. Interestingly though, only nucleus accumbens activity correlated with actual funding outcomes once crowdfunding deadlines had been reached several weeks later. This suggests that certain neural predictors for a sample may be more effective at forecasting population behavior than the behavioral choices made by the same sample.

Similarly, a study by Kühn et al. (2016) suggests that brain activations measured by fMRI in one group of participants can predict consumer behavior in an independent sample. In this study, researchers first examined brain activations in a small sample of participants (*N* = 18) while they viewed six different advertising posters for a single, new brand of chocolate bar. Participants also completed self-report liking judgments for each poster. The authors analyzed activations in *a priori* regions of interest that are related to key processes underlying consumer decision making (e.g., emotion, reward, memory, and working memory), such as the nucleus accumbens, medial orbitofrontal cortex, amygdala, hippocampus, inferior frontal gyrus, dorsomedial prefrontal cortex, dorsolateral prefrontal cortex and insula, and developed a model to predict people’s purchase behavior based on brain activation. This model successfully predicted which advertising poster would be most successful in the market place in an independent sample of over 60,000 supermarket shoppers. Critically, sales were accurately predicted by fMRI data, but not by self-report. Activity in the medial orbitofrontal cortex was found to be the most accurate predictor of future sales.

Likewise, Scholz et al. (2017) investigated the neural activity of participants in response to New York Times articles. During scanning, participants viewed 80 article headlines and abstracts and rated whether or not they would share the article with their friends on a popular online social media platform. Neural activity in the ventromedial prefrontal cortex was observed to correlate with the population-level data collating the number of online likes and shares that the articles subsequently received. Correlations exceeded those obtained for participants’ self-reported ratings of the articles, again providing evidence that sample data can predict population level marketing success.

Similar forecasting ability has also been observed using EEG. Dmochowski et al. (2014) observed that inter-participant correlation in the evoked EEG responses when watching popular broadcast television contents was predictive of the size of the audience of the contents in the population level. Interestingly, the inter-participant correlation in the EEG responses was more strongly correlated with the preference ratings in the population level, rather than the preference ratings from the participants’ themselves. Boksem and Smidts (2015) also used EEG to investigate neural responses to movie trailers. Beta and gamma oscillations in medial-frontal regions correlated with self-reported movie preferences, but also predicted wider commercial success of the movies in terms of box office takings.

Taken together, these results highlight that although EEG and neuroimaging are expensive techniques and it is not possible to collect data from everyone in the population, only small sample sizes may be needed in order to predict consumer behavior in an independent population when the sample is carefully chosen to represent the target population (Falk et al., 2013). Importantly, these forecasts have also been shown to be more accurate than those based on self-report measures, and in some cases more accurate than the actual behavior of the sample.

### Causality

It has been questioned whether physiological and neuroimaging measures aid in the identification of causal mechanisms underlying consumer behavior. In the previous section, we have shown that physiological and brain responses may help identify processes underlying consumers’ behavior, such as arousal, reward, attention, and pleasantness. However, it is important to note that while these emotional and cognitive reactions can elicit a physiological response, the reverse inference is not necessarily true. Physiological changes may result for many reasons other than as a direct response to marketing related stimuli, and so data should always be interpreted with caution. Nevertheless, when using careful experimental design, combining multiple physiological/brain measures, these measures can still be an effective tool in understanding processes and mechanisms underlying consumer behavior.

## Conclusion

The current paper gave an overview of several physiological and neuroscientific measures that allow the (indirect) measurement of various processes relevant in consumer behavior. Compared with self-report, these measures will often be unbiased and therefore provide a more complete and informative measure for marketing practioners. Further, they enable the highlighting of mechanisms underpinning consumer behavior, and therefore lead to an improved understanding of the relevant processes. As highlighted in many of the described studies, a combination of such measures might be best suited to unravel complex behavioral mechanisms. It is important to acknowledge that many of the findings of the reviewed studies represent correlational data from small, discrete samples of participants. Nevertheless, an emerging literature suggests that they not only help to explain the underlying mechanisms of consumer behavior, but also help predict consumers’ behavior at the population level. Together, these correlations between physiology, neurology and behavior, along with the introduction of new technology, will further aid our understanding of the mechanisms underlying consumer experience, potentially leading to improved predictions of consumer behavior and increasing the effectiveness of future marketing strategies. However, these neuromarketing strategies come with ethical implications to consumer privacy, autonomy, and control that will also need to be addressed to the satisfaction of the consumer in order for neuromarketing to achieve its full potential (Stanton et al., 2017). While these technologies are being perfected, well-designed experimental paradigms recording a range of self-report, physiological, and neuroscientific measures remain an effective way to improve and inform marketing strategies.

## Author Contributions

LB, JV, CW, MS, and LTB developed the structure and content of the manuscript. LB and JV drafted the manuscript. All authors provided edits and feedback.

## Conflict of Interest Statement

Co-author TR is founder of Experience Insight United Kingdom, who financially supported the paper. The remaining authors declare that the research was conducted in the absence of any commercial or financial relationships that could be construed as a potential conflict of interest.
